# Anti-Tumor Effect of Apatinib and Relevant Mechanisms in Liposarcoma

**DOI:** 10.3389/fonc.2021.739139

**Published:** 2021-11-18

**Authors:** Lixuan Cui, Liang Yan, Xiaoya Guan, Bin Dong, Min Zhao, Ang Lv, Daoning Liu, Zhen Wang, Faqiang Liu, Jianhui Wu, Xiuyun Tian, Chunyi Hao

**Affiliations:** ^1^ Key Laboratory of Carcinogenesis and Translational Research (Ministry of Education/Beijing), Department of Hepato-Pancreato-Biliary Surgery, Peking University Cancer Hospital and Institute, Beijing, China; ^2^ Key Laboratory of Carcinogenesis and Translational Research (Ministry of Education/Beijing), Central Laboratory, Peking University Cancer Hospital and Institute, Beijing, China; ^3^ Key Laboratory of Carcinogenesis and Translational Research (Ministry of Education/Beijing), Department of Pathology, Peking University Cancer Hospital and Institute, Beijing, China

**Keywords:** primary retroperitoneal liposarcoma, angiogenesis, apatinib, epirubicin, microvessel density

## Abstract

**Background:**

Primary retroperitoneal liposarcomas (RLPSs) are rare heterogeneous tumors for which there are few effective therapies. Certain anti-angiogenic tyrosine kinase inhibitors have demonstrated efficacy against various solid tumors. The aims of this study were to investigate the effect of Apatinib against retroperitoneal liposarcoma cells and its underlying mechanism and to explore the anti-tumor efficacy of a combination of Apatinib and Epirubicin.

**Methods:**

CD34 immunohistochemical staining was used to measure microvessel density (MVD) in 89 retroperitoneal liposarcoma tissues. We used CCK-8 cell proliferation, clone formation, Transwell migration, invasion assays and flow cytometry to evaluate the effects of Apatinib alone and the combination of Apatinib and Epirubicin on liposarcoma cells. High-throughput RNA sequencing and western-blotting was used to identify key differentially expressed genes (DEGs) in SW872 cell line after application of Apatinib. Murine patient-derived tumor xenograft (PDX) was established to assess the efficacy and safety of Apatinib monotherapy and the combination of Apatinib and Epirubicin in RLPS.

**Results:**

The microvessel density (MVD) varied widely among retroperitoneal liposarcoma tissues. Compared with the low-MVD group, the high-MVD group had poorer overall survival. Apatinib inhibited the liposarcoma cell proliferation, invasion and migration, increased the proportion of apoptosis, and induced G1 phase arrest. In addition, the combination of Apatinib and Epirubicin enhanced the foregoing inhibitory effects. High-throughput RNA sequencing showed that Apatinib downregulated the expression of TYMS and RRM2. Western blotting verified that Apatinib downregulated the TYMS/STAT3/PD-L1 pathway and inhibited liposarcoma proliferation by suppressing the RRM2/PI3K/AKT/mTOR pathway. In the murine PDX model of retroperitoneal liposarcoma, Apatinib and its combination with Epirubicin significantly inhibited microvessel formation and repressed tumor growth safely and effectively.

**Conclusions:**

Apatinib and its combination with Epirubicin showed strong efficacy against liposarcoma both *in vitro* and *in vivo*. Apatinib might inhibit liposarcoma cell proliferation through the RRM2/PI3K/AKT/mTOR signaling pathway and downregulate PD-L1 *via* the TYMS/STAT3 signaling pathway.

## Introduction

Retroperitoneal soft tissue sarcomas (RPS) are rare tumors with an incidence of 0.5-1 per 100,000 residents. Liposarcoma is the most common subtype which originates from adipose tissue, accounting for 45% of all RPS (1, 2). At present, the main treatment for retroperitoneal liposarcoma (RLPS) is still limited to surgery. Chemotherapy has controversial therapeutic efficacy against RLPS (3, 4). Further studies are needed to identify novel therapeutic regimens for RLPS.

Angiogenesis plays an important role in tumor development and progression. Compared with normal tissues, tumor tissues require abundant new blood vessels to meet their growth and metastasis requirements (5). Unlike the traditional treatment regimens focusing on tumor cells, anti-angiogenic therapy targets angiogenesis (6). Inhibition of tumor angiogenesis reduces the blood supply to the tumor tissue, thereby inhibiting tumor growth and proliferation (7). The receptor tyrosine kinase-mediated signaling pathway is the most comprehensively elucidated mechanism involved in angiogenesis. This pathway comprises multiple receptor tyrosine kinases such as VEGFR, PDGFR, FGFR, and Tie-2 (8), so targeting receptor tyrosine kinase is a potential anti-angiogenesis strategy.

Apatinib, also known as YN968D1, is a novel small molecule tyrosine kinase inhibitor of vascular endothelial growth factor receptor–2(VEGFR-2). It can also inhibit c-Kit and Ret (9). Apatinib has been approved by China Food and Drug Administration for the treatment of advanced metastatic gastric cancer. It also has shown promising antitumor efficacy in breast cancer, non-small-cell lung cancer (NSCLC) and hepatocellular carcinoma (HCC) (10).To date, only a few case reports have confirmed that Apatinib could be effective against certain subtypes of liposarcomas (11, 12). However, there is no preclinical study evaluating the safety or efficacy of Apatinib alone or in combination with cytotoxic chemotherapy drugs in liposarcoma treatment. Moreover, mechanisms by which Apatinib inhibits liposarcoma proliferation remains unclear.

Epirubicin is widely used as a chemotherapeutic agent for the treatment of various tumors such as breast cancer, gastric cancer, and soft tissue sarcoma (13). Only a few drugs are determined to be effective in the treatment of soft tissue sarcomas, wherein Doxorubicin, Epirubicin and Ifosfamide are the only chemotherapy drugs with a response rate of over 20% (14). In particular, Epirubicin is a derivative of doxorubicin, which differs from Doxorubicin in the configurations of the 4 ‘position of the hydroxyl group (15). Compared with Doxorubicin, Epirubicin has lower cardiotoxicity (13, 16). Epirubicin inhibits tumor growth through inhibiting the activity of topoisomerase II, thus generating oxygen and free radicals and then interfering with the replication of DNA and RNA.

In the present study, we measured microvessel density (MVD) in retroperitoneal liposarcoma tissues, assessed the prognostic value of MVD for RLPS, and evaluated the anti-tumor efficacy of Apatinib alone or in combination with Epirubicin in liposarcoma cells. RNA-seq identified differentially expressed genes (DEGs) after Apatinib treatment. A murine RLPS patient-derived tumor xenograft (PDX) model was established to validate the safety and efficacy of Apatinib alone or its combination with Epirubicin. This study could provide preclinical data for the potential application of Apatinib in liposarcoma therapy.

## Materials and Methods

### Patients and Samples

Retroperitoneal liposarcoma tissues were collected from 89 patients who had undergone surgical resection at Peking University Cancer Hospital between 2010 and 2019. The median follow-up time was 38.8 months (range: 1.4-104.4months). Patients who received chemotherapy or radiation therapy were excluded from this study. The present study was approved by the institutional review board of Peking University Cancer Hospital (No. 2021KT43). All patients signed written informed consent. Details of the clinicopathological characteristics of the patients are listed in **Table 1**.

**Table 1:** Association between microvessel density (MVD) and clinicopathological characteristics of 89 patients with retroperitoneal liposarcoma.

Clinicopathological Feature	Total	MVD		P value
		High (%)	Low (%)	
**Gender**				
Male	50	27 (54.0)	23 (46.0)	0.330
Female	39	17 (43.6)	22 (56.4)	
**Age**				
< 60	54	28 (51.8)	26 (48.1)	0.572
≥ 60	35	16 (45.7)	19 (54.3)	
**Tumor size**				
<30	65	35 (53.8)	30 (46.2)	0.171
≥30	24	9 (37.5)	15 (62.5)	
**Grade**				
Low (G1)	20	10 (50.0)	10 (50.0)	0.954
High (G2, G3)	69	34 (49.3)	35 (50.7)	
**Pathological** **classification**				
DDLPS	61	30 (49.2)	31 (51.7)	0.671
WDLPS	20	9 (45.0)	11 (55.0)	
PLPS	6	3 (50.0)	3 (50.0)	
MLPS	2	2 (100.0)	0 (0.0)	
**Multifocality**				
No	53	28 (52.8)	25 (47.2)	0.464
Yes	36	16 (44.4)	20 (55.6)	
**Necrosis**				
No	58	24 (41.4)	34 (58.6)	**0.038**
Yes	31	20 (64.5)	11 (35.5)	
**Recurrence**				
No	48	25 (52.1)	23 (47.9)	0.589
Yes	41	19 (46.3)	22 (53.7)	

DDLPS: Dedifferentiated liposarcoma; WDLPS: Well-differentiated liposarcoma; PLPS: Pleomorphic liposarcoma; MLPS: Myxoid liposarcoma; Bold font: significant statistical difference.

### Reagents, Cell Culture and Cell Viability Assay

The 93T449, 94T778 and SW872 cell lines were purchased from ATCC (American Type Culture Collection, Manassas, VA, USA). The cells were cultured in RPMI 1640 medium supplemented with 10% (v/v) FBS, penicillin, and streptomycin in a 37°C humidified incubator under 5% CO_2_ atmosphere. Intact exponential phase cells were used in the subsequent experiments.

Anti-CD34 (No. ab81289), anti-RRM2(No. ab108995), and anti-TYMS (No. ab57653) were obtained from Abcam, Cambridge, UK. Anti-STAT3(No. 4904T), anti-p-STAT3(No.9145T),anti-AKT(No.4691T),anti-p-AKT(No.4060T),anti-mTOR(No.2983T),anti-p-mTOR(No.5536T),anti-PD-L1(No.13684S),anti-PI3K(No. 4249T), and anti-p-PI3K(No. 17366S) were acquired from Cell Signaling Technology, Danvers, MA, USA. Anti-β-actin (No. A4552) was purchased from Sigma-Aldrich Co., St. Louis, MO, USA. Anti-Ki67 was purchased from Beijing Zhongshan Jinqiao Biotechnology Co., Ltd, Beijing, China. Apatinib (No. S5248) and Epirubicin HCl (No. S1223) were obtained from Selleck Chemicals LLC, Houston, TX, USA.

Cell viability was evaluated by CCK8 assay (No.CK04-500T; Shanghai Dojindo Laboratories, Shanghai, China). Before the IC50 assays, the 93T449, 94T778, and SW872 cell lines were inoculated in 96-well plates at the density of 4,000/well and 4,500/well. Various concentrations of the drugs were added, and the cells were incubated for 48 h. Absorbance (OD) was measured in a microplate reader at 450nm. For the cell proliferation experiment, the 93T449 and SW872 inoculation densities were 3,000/well and 2,000/well, respectively. Various concentrations of drugs were added, and the cells were incubated for 96 h. OD_450_ were measured in the microplate reader.

### Apoptosis and Cell Cycle Analysis

For the apoptosis analysis, the cells were inoculated in a six-well plate at 4×10^5^/well. Aptinib, Epirubicin or a combination of two drugs were added to the wells and the cells were incubated for 24 h. The cells were then stained with propidium iodide (PI) and Annexin-FITC. A flow cytometer (CytoFLEX; Beckman Coulter, Brea, CA, USA) was used to assess the apoptosis. FITC-PI double staining indicated terminal apoptosis, FITC single staining indicated early apoptosis, and PI single staining indicated cell death.

After the cells were treated with various drug concentrations for 24 h, they were digested by trypsin and washed with phosphate-buffered saline (PBS). The cells were fixed with cold 75% (v/v) ethanol for 24 h and stained with 300μL PI/RNase Staining Buffer (No. 550825; BD Biosciences, Franklin Lakes, NJ, USA) in a dark room for 15 min. The cell cycle was measured by flow cytometer (Accuri C6; BD Biosciences, Franklin Lakes, NJ, USA). Cell cycle data were analyzed with ModFit v. 4.1.

### Invasion and Migration Analysis

Transwell chamber (No. 3422; Corning, Corning, NY, USA) was used for the migration assay. The Matrigel invasion chamber (No. 354480, Corning, Corning, NY, USA) was used for invasion assay. The chamber used for invasion assay is pre-packaged with Matrigel. The chamber was hydrated with serum-free medium for at least 2 h. Then 200 μL serum-free medium (for migration assay) or 500 μL serum-free medium (for invasion assay) containing cells (A total of 1×10^5^ 93T449 cells or 5×10^4^ SW872 cells) that have been incubated with different drugs for 24 h to the upper chamber. Then 500μL RPMI-1640 medium containing 20% (v/v) FBS was added to the lower chambers. After 24 h of incubation for migration and 48 h for invasion at 37°C, the cells in the upper chamber were removed. 4% (v/v) paraformaldehyde was added to fix the cells that had invaded/migrated and 0.1% (w/v) crystal violet was used to stain the cells. The cells penetrating the membrane were counted by ImageJ (NIH, Bethesda, MD, USA) at 100× magnification.

### RNA-Seq and Data Analysis

SW872 cells were treated with Apatinib (18μM) for 24 h. The RNAs of the control group and experimental group were stored in TRIzol reagent at -80° C. Then mRNA extraction and RNA sequencing were performed by Beijing Mygenostics Co. Ltd., Beijing, China. A Bioanalyzer 2100 System (Agilent Technologies, Santa Clara, CA, USA) was used to assay RNA integrity. An AMPureXP system (BeckmanCoulter, Brea, CA, USA) was used to perform PCR on the products. Quality-tested library preparations were sequenced on the Illumina Novaseq platform (Illumina, San Diego, CA, USA). There were three biological replicates per condition. Differential expression analysis of both groups was performed using DESeq2R v. 1.20.0. The Benjamini-Hochberg multiple hypothesis test correction was performed to obtain the false discovery rate (FDR). The criteria used to select the DEGs were |log2(FoldChange)| > 0.5 and FDR < 0.05. When log2(FoldChange) > 0, the gene was considered upregulated. When log2(FoldChange) < 0, the gene was considered downregulated. profiler package in R (R Core Team, Vienna, Austria) was used for GO function and KEGG pathway enrichment analyses of the DEGs. Histograms and bubble graphs were plotted with the ggplot2 package in R v. 3.4.3.

### Western Blot Analysis

When the cells proliferate to 60% of the area of the culture plate, add different concentrations of drugs and incubate for 24 hours at 37° C, then discard the drug solution and culture medium, and extract cell proteins for subsequent Western blot experiments. Total proteins were collected from cells lysed with radioimmunoprecipitation assay (RIPA) lysate (No. 9806; Cell Signaling Technology, Danvers, MA, USA). Cellular proteins were separated by 6% or 8% sodium dodecyl sulfate-polyacrylamide gel electrophoresis (SDS-PAGE), depending on their molecular weight. The proteins were then transferred to a polyvinylidene fluoride (PDVF) membrane which was then blocked with 5% (w/v) skim milk for 1 h. The PDVF membrane was incubated with primary antibody at 4° C overnight. After returning to room temperature, the membrane was incubated with the corresponding secondary antibody for 1h.

The chemiluminescence reaction was developed with Immobilon Western HRP Substrate Luminal Reagent (No. WBKLS0500; EMD Millipore, Billerica, MA, USA). The protein bands was detected with an enhanced chemiluminescence (ECL) detection system (Amersham Imager 600; General Electric Co., Boston, MA, USA).

### Immunohistochemistry Staining

Paraffin tissue sections were baked at 72°C for 1 h, dewaxed with xylene and hydrated with gradient ethanol. Endogenous hydrogen peroxide was consumed with a 3% (v/v) hydrogen peroxide solution. Anti-RRM2 (1:800), anti-CD34 (1:200) were treated with citric acid (pH 6.0) under microwave for 10 min. Anti-TYMS (1:100) and anti-PD-L1 were treated with EDTA (pH 9.0). Anti-Ki67 was treated with EDTA (pH 8.0) under high temperature and pressure for 2.5 min. After cooling to room temperature, the tissues were sealed with goat serum for 1 h. And then primary antibody was added, and the sections were incubated at 4° C overnight. The sections were then removed and restored to room temperature on the second day. Secondary antibody was added, and the sections were incubated at room temperature for 30 min. The sections were then stained with diaminobenzidine (DAB) and hematoxylin. An ethanol concentration gradient and xylene were used for dehydration and clarifying the sections, respectively. Three pathologists independently enumerated the positive cells in the sections and the staining intensity in the sections.

### Microvessel Density (MVD) Analysis

Yellow-brown endothelial cells or cell clusters subjected to immunostaining were regarded as a single countable microvessel. Five “hot spots” with the highest MVD were selected under the 100× magnification. NIS-Elements Viewer (Nikon, Chiyoda, Japan) was used to calculate the number of microvessels in the corresponding field of view under the 200×magnification (17). MVD was defined as the number of microvessels per field divided by the field area. The single visual field area in NIS-Elements Viewer software is 0.3250 mm². The MVD was independently counted by three pathologists blinded to patient status.

### Patient-Derived Tumor Xenograft

Twenty mice aged 4-5 weeks and weighing 18-19g were selected for experiments. The tumor tissue implanted in mice was derived from a case of retroperitoneal liposarcoma (P0) that can be passaged stably. The case of the P0 generation harvested from a 54 years old male who underwent retroperitoneal sarcoma resection on August 22, 2019 in our hospital and was pathologically diagnosed as dedifferentiated liposarcoma after surgery. The results of postoperative pathological immunohistochemistry are as follows: β-catenin (–), CD34(+), CK7 (–), Desmin (–), Ki67(60%+), SMA (–), STAT6 (–), S100 (–), CD6(+), MDM2(+), MyoD1 (–), Myoglobin (–), CD56(+).

Then the primary PDX tissue was implanted in the right sides of the backs of the mice. After P1 generation was transplanted into mice, the tumor size was measured every 3 days. When the tumor volume was close to 1000mm^3^, the mice were sacrificed, and the tumor tissues were taken out for passage and also saved in the liquid nitrogen for future use. The establishment of P2 generation mice was the same as that of P1 generation mice. When the tumor volume of P2 generation mice was close to 1000mm^3^, the mice were sacrificed. The tumor tissues are taken out and cut into small tissue pieces of about 5mm^3^, and they were transplanted in 24 mice to establish P3 generation mouse model. In our study, 20 P3 generation PDX models were successfully established and applied in subsequent drug experiments.

When the tumor volumes reached 100-150 mm^3^, the mice were divided into groups by the random number table method. There were five mice per group. The tumor size was measured every 3 d. The tumor volume was calculated by multiplying the long diameter by the square of the short diameter divided by 2. Each mouse in the Apatinib group was administered oral Apatinib (100 mg/kg) daily. Each mouse in the Epirubicin group was administered intravenous Epirubicin (2.5mg/kg) weekly on days 1, 8, and 15. The Apatinib was suspended in 0.5% CMC-NA. The present study was approved by the Animal Ethics Committee of Peking University (No. EAEC2018-06).

On the 22^nd^ day of the experiment, venous blood was collected from the orbit, and the plasma was obtained by static centrifugation. The biochemical indices were measured with a Mindray automatic biochemical analyzer (Shenzhen Mindray Co. Ltd., Shenzhen, China), and the mice were sacrificed.

### Statistical Analysis

The correlation between MVD and clinicopathologic features in patients with RLPS was evaluated with two-tailed χ^2^ and Fisher’s exact tests.

A Kaplan–Meier survival analysis and Log-rank test were used to analyze the correlations between MVD and patient prognosis. An independent sample t-test was used to detect the differences between the two sample groups. The results were expressed as means ± SD. A Cox proportional hazard regression model was used for univariate and multivariate survival analyses to identify independent risk factor affecting overall survival (OS). When the P value was < 0.05, the result was considered significant. Data were analyzed with SPSS v. 22.0 (IBM Corp., Armonk, NY, USA).

The combination index (CI) of the Apatinib and Epirubicin was calculated by Chou-Talalay method with Compusyn. When CI was < 1, the interaction between the drugs is synergistic. When CI was > 1, the interaction between the drugs is antagonistic (18).

## Results

### MVD Patterns and Clinicopathological Features in Liposarcoma

**Figure 1:**
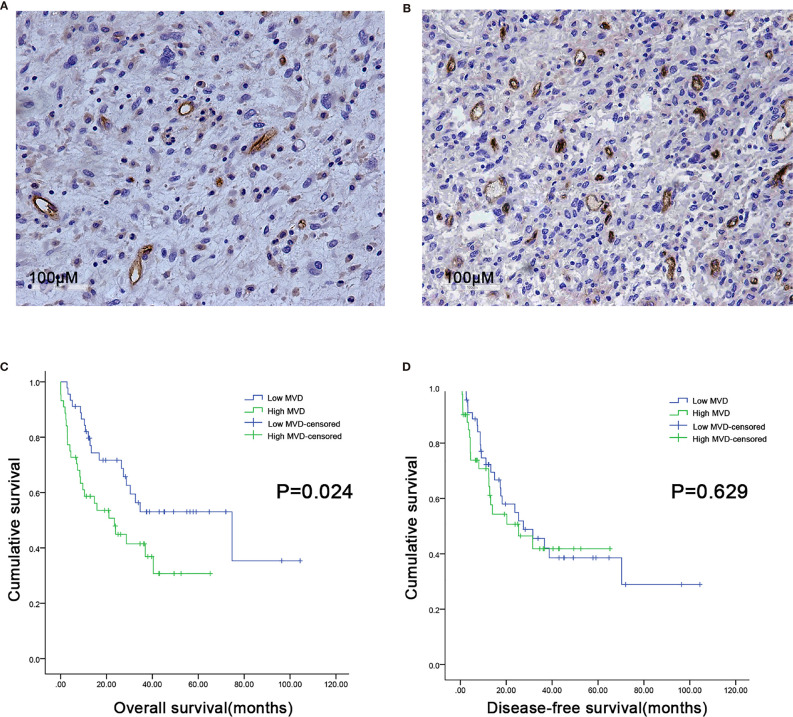
Microvessel distribution in retroperitoneal liposarcoma tissues. **(A)** Representative images of RLPS tissues with low MVD. There are seven microvessels in the field. MVD = 21.9/mm^2^. **(B)** Representative images of RLPS tissues with high MVD. There are 37 microvessels in the field. MVD = 113.8/mm^2^. ×200 magnification. **(C)** Kaplan–Meier survival analysis and Log–rank tests revealed that high MVD was correlated with poor OS (P = 0.024). **(D)** MVD was not related to disease-free survival (DFS) in RLPS patients (P = 0.629).

We used CD34 staining to measure microvessel density in 89 retroperitoneal liposarcoma patients. The median MVD was 56.9/mm^2^ (range: 12.5–225.0/mm^2^). Microvascular density varied greatly in retroperitoneal liposarcoma. Typical microvascular staining is shown in **Figure 1A, B**. The cutoff value for categorical MVD evaluation was predefined as the median microvessel count. We found a significant correlation between MVD and necrosis (P = 0.038) (**Table 1**). A Kaplan–Meier survival analysis and Log-rank test revealed that the high MVD was correlated with poor overall survival (OS) (P=0.024). The median OS time of the high-MVD group was 23.5 months (95% CI; 10.9–36.1 months), and the median OS time of the low-MVD group was 74.7 months (95% CI; 13.9–135.7 months) (**Figure 1C**). There was no significant difference in disease-free survival (DFS) between the high-MVD and low-MVD groups (P = 0.629) (**Figure 1D**). A multivariate analysis revealed that MVD was an independent risk factor for OS (HR:1.974, 95%CI:1.081-3.607; P=0.001) (**Table 2**).

**Table 2:** Univariate and multivariate analyses of independent risk factors for OS in retroperitoneal liposarcoma patients.

Clinicopathological Feature	Univariate analysis		Multivariate analysis	
	HR (95% CI)	P value	HR (95% CI)	P value
**MVD**				
Low	1		1	
High	1.974 (1.081–3.607)	**0.027**	3.334 (1.630–6.819)	**0.001**
**Gender**				
Male	1			
Female	1.438 (0.800–2.585)	0.224		
**Age**				
≤ 60	1			
> 60	1.203 (0.663–2.185)	0.543		
**Tumor size(cm)**				
≤ 30	1		1	
> 30	2.352 (1.280–4.324)	**0.006**	3.765 (1.829–7.747)	**<0.001**
**Grade**				
Low (G1)	1		1	
High (G2, G3)	2.422 (1.016–5.777)	**0.046**	2.245 (0.937–5.377)	0.070
**Pathological classification**		0.142		
DDLPS	1			
WDLPS	0.341 (0.134–0.871)	0.024		
PLPS	0.690 (0.212–2.245)	0.538		
MLPS	1.425 (0.193–10.496)	0.728		
**Multifocality**				
No	1			
Yes	1.175 (0.650–2.123)	0.594		
**Necrosis**				
No	1		1	
Yes	1.671 (0.925–3.019)	0.089	1.457 (0.796–2.667)	0.222
**Primary/Recurrent**				
Primary	1			
Recurrent	1.227 (0.679–2.218)	0.497		

DDLPS: Dedifferentiated liposarcoma; WDLPS: Well-differentiated liposarcoma; PLPS: Pleomorphic liposarcoma; MLPS: Myxoid liposarcoma; HR: Hazard ratio; CI: Confidence interval; MVD: Microvessel density; Bold font: significant statistical difference.

### Combination of Apatinib and Epirubicin Inhibits Liposarcoma Cell Proliferation

**Figure 2:**
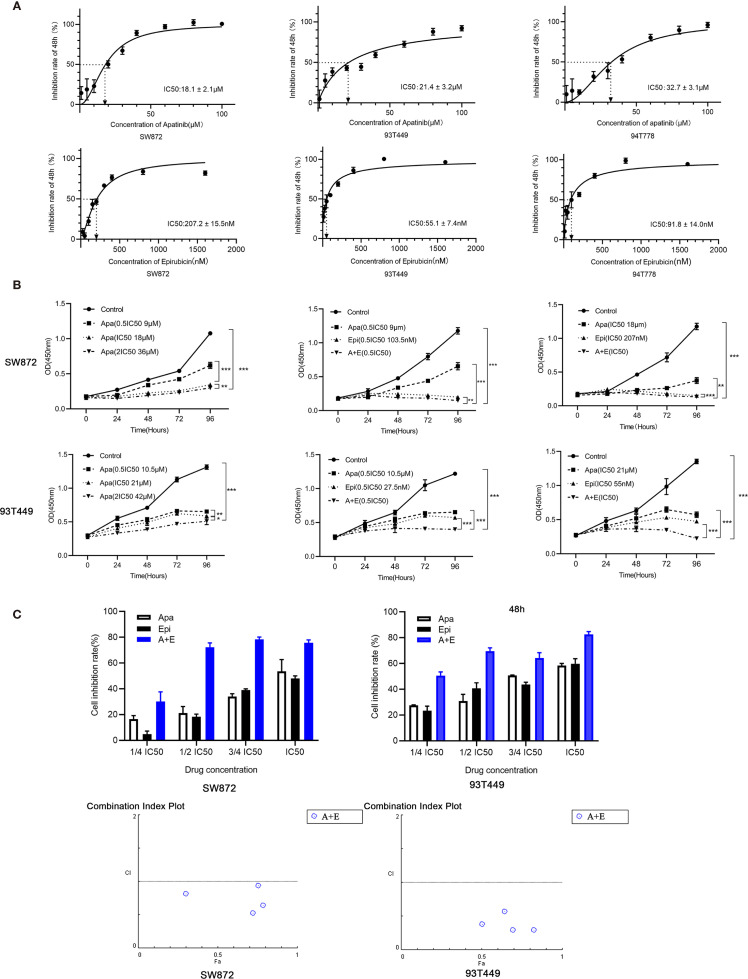
Apatinib and its combination with Epirubicin inhibited proliferation of RLPS cells. **(A)** CCK-8 assay revealed that the IC50 of Apatinib were 18.1 ± 2.1μM for SW872 cells, 21.4 ± 3.2 μM for 93T449 cells, and 32.7 ± 3.1 μM for 94T778 cells and the IC50 of Epirubcin were 207.2 ± 15.5 nM for SW872 cells, 55.1 ± 7.4 nM for 93T449 cells, and 91.8± 14.0 nM for 94T778 cells. **(B)** Apatinib alone and its combination with Epirubicin inhibited RLPS cell proliferation. **(C)** Combination of Apatinib and Epirubicin synergistically inhibited liposarcoma proliferation. Fa: fractional activity (efficacy); CI: combination index; (^*^P < 0.05, ^**^P < 0.01, ^***^P < 0.001).

The cells were cultured with different concentrations of Apatinib(1μM, 5μM, 10μM, 20μM, 30μM, 40μM, 60μM, 80μM, 100μM) for 48h. The IC50 of Apatinib was 18.13 ± 2.04μM for SW872 cells, and 21.44 ± 3.11μM for 93T449 cells and 32.69 ± 3.12μM for 94T778 (**Figure 2A**). Based on the sensitivity for Apatinib, SW872 and 93T449 were used for further investigation subsequent experiments. CCK8 assay revealed that the growth of liposarcoma cells were inhibited by Apatinib monotherapy and in combination with Epirubicin in a time-dependent and dose-dependent manner (**Figure 2B**). Lower drug-concentration (¼ IC50-IC50) treatment was used to evaluated the synergistic inhibition effect of Apatinib and Epirubicin. All the combination index values were less than 1, indicating that these two drugs had synergistic inhibition effect on cell proliferation (**Table 3** and **Figure 2C**).

**Table 3:** Synergistic inhibition of Apatinib and Epirubicin in liposarcoma cells.

Drug concentration	SW872		93T449	
	Fa	CI	Fa	CI
¼ IC50	0.30	0.81	0.51	0.38
½ IC50	0.72	0.52	0.70	0.30
¾ IC50	0.78	0.64	0.64	0.57
IC50	0.76	0.93	0.82	0.29

Fa, Fraction affected; CI, Combination index.

### Combination of Apatinib and Epirubicin Inhibits Liposarcoma Cell Migration and Invasion

**Figure 3:**
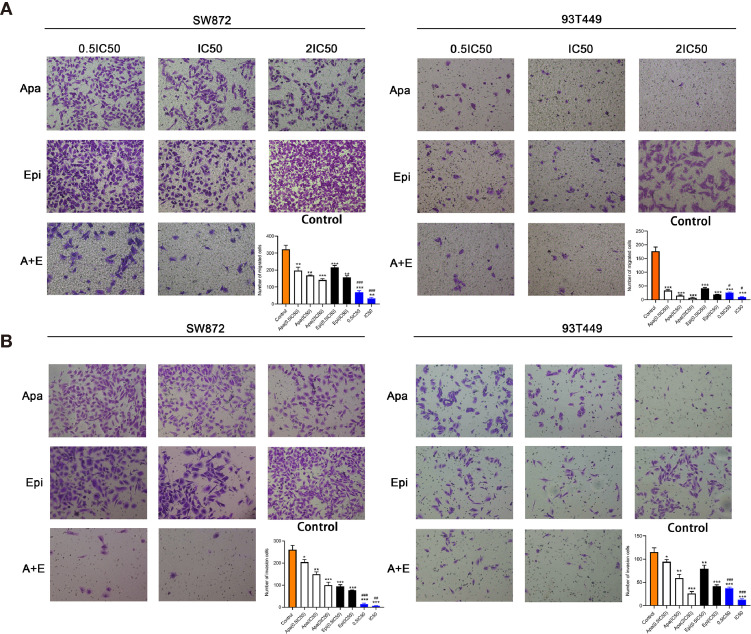
Apatinib and its combination with Epirubicin inhibited migration and invasion of SW872 and 93T449 cells. **(A)** Transwell assays revealed that Apatinib and its combination with Epirubicin reduced the number of migrating liposarcoma cells. **(B)** Transwell assays revealed that Apatinib alone and its combination with Epirubicin reduced the number of invading liposarcoma cells. ×100 magnification. ^*^/^**^/^***^ for comparison between treatment and control groups, ^*^P < 0.05, ^**^P < 0.01, ^***^P < 0.001; ^#^/^##^/^/###^ for comparison between Apatinib group and combination group, ^#^P < 0.05, ^##^P < 0.01, ^###^P < 0.001; Apa, Apatinib; Epi, Epirubicin; A+E, combination of Apa and Epi.

Transwell assays were performed to determine whether Apatinib affects liposarcoma cell invasion and migration. **Figure 3** shows that Apatinib alone and its combination with Epirubicin significantly reduced SW872 and 93T449 cell migration and invasion. **Figure 3A** indicates that there were 321.7 ± 24.7 migrated SW872 cells per field in the control group but only 168.0 ± 3.0 migrated SW872 cells per field after Apatinib (18 μM, IC50) treatment (P < 0.01). After the combination treatment, there were only 32.7 ± 5.5 migrated SW872 cells per field (P < 0.01). The number of migrated SW872 cells was significantly lower in the combination group than the Apatinib group (P < 0.001). **Figure 3B** shows that there were 149.7 ± 10.2 invaded SW872 cells per field (P < 0.01) in the Apatinib group (18 μM, IC50), 76.7 ± 1.5 invaded SW872 cells per field (P < 0.001) in the Epirubicin group (207 nM, IC50), and 6.0 ± 1.7 invaded SW872 cells per field (P < 0.001) in the combination group (IC50). In contrast, there were 260.0 ± 20.0 invaded SW872 cells per field in the control group. The number of migrated SW872 cells was significantly lower in the combination group than the Apatinib group (P = 0.001). Similar results were obtained for the 93T449 cell line. The inhibition effect of 0.5 IC50 combination concentration was similar to that of IC50 combination concentration.

### Combination of Apatinib and Epirubicin Induces Apoptosis and Cell-Cycle Arrest

**Figure 4:**
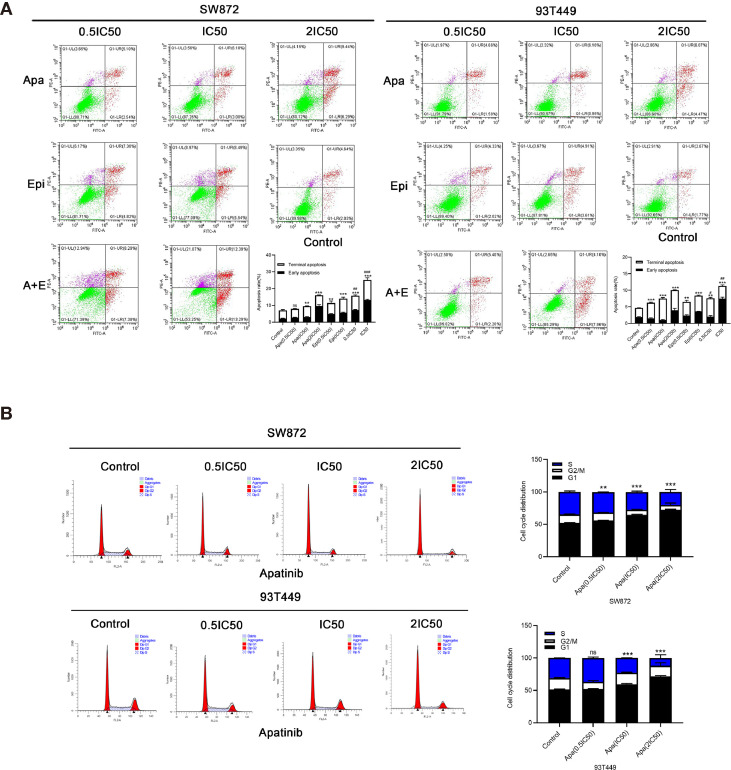
Effects of Apatinib and its combination with Epirubicin on apoptosis and cell cycle of liposarcoma cells. **(A)** Apatinib increased the rate of apoptosis, and the combination of Apatinib and Epirubicin synergically induced apoptosis. **(B)** Apatinib induced G0/G1 phase cell arrest in liposarcoma cells. ^**^/^***^ for comparison between each treatment group and control group, ^**^P < 0.01, ^***^P < 0.001; ^#^/^##^/^###^ for comparison between Apatinib group and combination group, ^#^P < 0.05, ^##^P < 0.01, ^###^P < 0.001; ns: non-significant; Apa, Apatinib; Epi, Epirubicin; A+E, combination of Apa and Epi.

To determine the effect of Apatinib on the liposarcoma cell cycle, we analyzed the cells by flow cytometry after Annexin-FITC/PI double staining. **Figure 4A** shows that apoptosis rate was significantly higher in the Apatinib group than the control group (P < 0.01) and the percentage of apoptosis was concentration-dependent. The combination of Apatinib and Epirubicin can significantly increase the proportion of total apoptosis compared with Apatinib alone (P<0.01).

We used flow cytometry to calculate the ratios of each cell cycle phase in liposarcoma cells treated with Apatinib for 24h. **Figure 4B** shows that Apatinib induced the accumulation of the SW872 and 93T449 cells in G0/G1 phase, and reduced the ratio of these cells in G2/M and S phase. These findings indicated that Apatinib may inhibit the proliferation of liposarcoma cells by promoting apoptosis and G0/G1-phase cell cycle arrest.

### Apatinib Downregulates the RRM2/PI3K/AKT/mTOR and TYMS/STAT3/PD-L1 Signal Pathway

**Figure 5:**
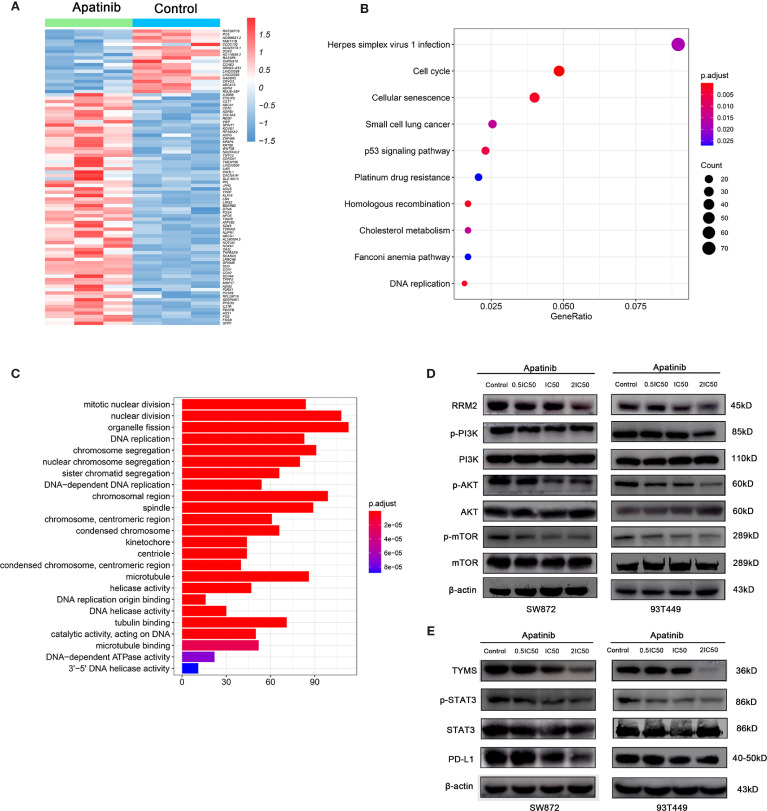
Apatinib inhibited RRM2 and TYMS-related pathways in liposarcoma. **(A)** Heatmap of top representative DEGs associated with Apatinib treatment in SW872 cell lines. **(B)** KEGG pathway enrichment analysis indicated that Apatinib could inhibit liposarcoma proliferation through p53 signaling, DNA replication, and other important signaling pathways. **(C)** GO analysis showed that Apatinib application affected mainly organelle fission, DNA replication, mitosis, cell cycle, and other important processes. **(D)** Western blotting (WB) showed that Apatinib downregulates RRM2, p-PI3K, p-AKT, and p-mTOR expression in liposarcoma cells compared with control group. **(E)** WB indicating that Apatinib downregulates TYMS, p-STAT3, and PD-L1expression in liposarcoma cells compared with control group.

To elucidate the mechanisms by which Apatinib inhibited liposarcoma cells, we used RNA-seq to evaluate relative genetic changes in the SW872 cells after being treated with Apatinib for 24 hours, and found 2,038 DEGs, including 1,059 significantly downregulated genes and 979 significantly upregulated genes. A representative heatmap of the DEGs is shown in **Figure 5A**. Top ten significantly up-regulated and down-regulated genes in SW872 cell line after apatinib application was shown in **Table S1**. The output of the KEGG pathway enrichment analysis shown in **Figure 5B** suggests that Apatinib might inhibit SW872 cell proliferation through p53, DNA replication, and other important signaling pathways. GO enrichment analysis was performed on the genes that were significantly altered after Apatinib application. **Figure 5C** shows that Apatinib affects mainly organelle fission, DNA replication, mitosis, cell cycle, and other important processes.

We then focused on the changes in gene expression that are closely related to the onset and progression of liposarcoma. **Table 4** shows that TYMS and RRM2 mRNAs were significantly downregulated after Apatinib treatment. Subsequent western blotting verified the changes in gene expression and downstream signaling pathways of TYMS and RRM2.

**Table 4:** High-throughput RNA sequencing of key gene expression in target therapy of liposarcoma.

Gene	log2 FoldChange	P value	FDR
TYMS	-0.52162	**0.007577**	0.045288
RRM2	-0.86155	**8.24E-07**	4.52E-05
CDK4	0.11233	0.47297	0.68214
MDM2	-0.29832	0.062491	0.19214

FDR: False discovery rate; Bold font: significant statistical difference.

To determine whether Apatinib modulates liposarcoma cell proliferation through the RRM2/PI3K/AKT pathway, we used WB to measure the protein levels of RRM2, PI3K, p-PI3K, AKT, p-AKT, mTOR, and p-mTOR. **Figure 5D** shows that the levels of PI3K, AKT and mTOR did not change whereas the levels of RRM2, p-AKT, p-mTOR, and p-PI3K decreased with the increasing Apatinib concentration. These findings indicated that Apatinib might affect the proliferation and apoptosis by inactivation of the RRM2/PI3K/AKT/mTOR signal pathway.

Previous literature stated that TKI can alter PD-L1 expression. PD-L1 is a key downstream molecule in the TYMS/STAT3 signaling pathway. Here, we used WB to examine the effects of Apatinib on TYMS/STAT3 signaling. **Figure 5E** shows that Apatinib downregulated PD-L1 and pSTAT3 without changing STAT3 level. Therefore, Apatinib might inhibit STAT3 and then downregulate PD-L1 expression in liposarcoma cells.

### Apatinib and Its Combination With Epirubicin Inhibited Growth of Liposarcoma *In Vivo*

**Figure 6:**
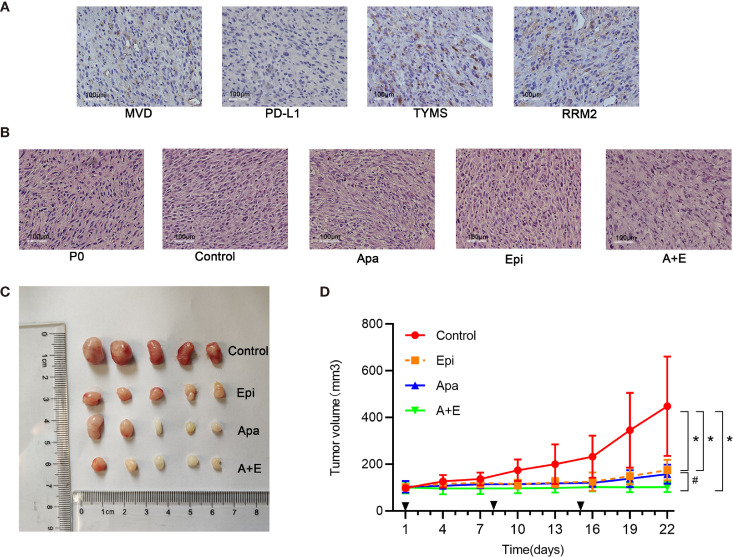
Apatinib and its combination with Epirubicin inhibited liposarcoma proliferation *in vivo*. **(A)** RRM2, TYMS, and PD-L1 expression and microvessel distribution in primary RLPS tissue. ×200 magnification. **(B)** Typical H&E staining of tumor sections in each group and primary RLPS tissue. ×200 magnification. **(C)** Macroscopic examination of resected tumors in each group. **(D)** Tumor growth curves for control group, single-drug group, and combination group. ^*^for comparison between administration and control groups; ^*^P < 0.05; ^#^for comparison between Apatinib group and combination group; ^#^P < 0.05; Apa, Apatinib; Epi, Epirubicin; A+E, combination of Apa and Epi.

The primary tumor tissue (P0) used to establish RLPS PDX model originated from a male patient with retroperitoneal dedifferentiated liposarcoma who underwent resection surgery on August 22, 2019. TYMS and RRM2 were positive while PD-L1 was negative in the tissues of the P0 generation. The microvessel density was 64.6/mm^2^ (**Figure 6A**). PDX tumor tissue was stably passaged to P3 for the experiment, with 5 mice in each group. Apatinib (100mg/kg) and 0.5% CMC-Na were given in the Apatinib group and control group, respectively. Epirubicin(2.5mg/kg) were given in the Epirubcin group. Apatinib (100mg/kg) and Epirubicin(2.5mg/kg) were given in the combination group as described above. The mice were sacrificed after 21 d and their transplanted tumors were dissected. Hematoxylin & eosin (H&E) staining of tumor tissue from the control group and experimental group is shown in the **Figure 6B**. Macroscopic examination of resected tumors in each group was shown in **Figure 6C**. The changes in mouse tumor volume over 21 d treatment were recorded (**Figure 6D**). In the control group, the average tumor volume was 448.0 ± 212.8 mm^3^. PDX growth was inhibited in the Epirubicin (175.1 ± 44.2 mm^3^; P = 0.044), Apatinib (157.6 ± 40.8 mm^3^; P = 0.037), and combination (102.5 ± 21.3 mm^3^; P = 0.022) groups. Compared with the Epirubicin group and Apatinib group, the tumor volumes in the combination group were significantly lower (P = 0.011; P = 0.036). Thus, the antitumor efficacy of the combination group was stronger than those of the monotherapy groups. This finding was consistent with the results of the *in vitro* experiments mentioned above.

**Figure 7:**
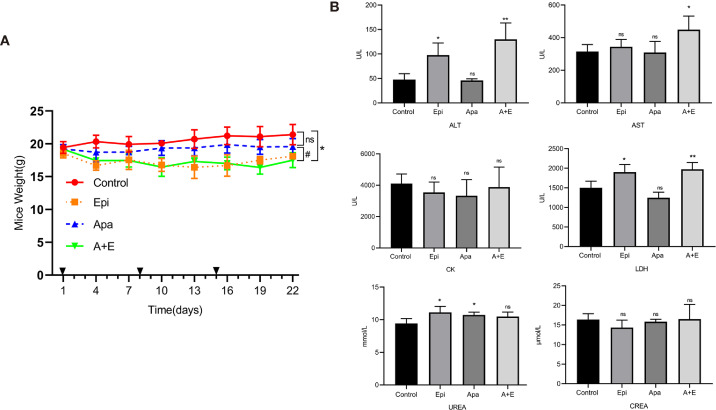
Safety of Apatinib and its combination with Epirubicin in retroperitoneal liposarcoma PDX. **(A)** Body weight changes in each group. Compared with Epirubicin group and combination group, Apatinib had less effect on the body weight of mice. **(B)** Serum ALT, AST, CK, LDH, UREA, and CREA levels in each group. Compared with the control group, ALT (P = 0.011), UREA (P = 0.026), and LDH (P =0.021) increased in the Epirubicin group, UREA (P = 0.023) increased in the Apatinib group, and ALT (P = 0.011), AST (P = 0.029), and LDH (P = 0.008) increased in the combination group. ^*^/^**^ for comparison between treatment group and control group; ^*^P < 0.05, ^**^P < 0.01; ns: non-significant; Apa, Apatinib; Epi, Epirubicin; A+E, combination of Apa and Epi.

Using the RLPS PDX model, we also evaluated the safety of Apatinib alone and its combination with Epirubicin. No mice died in any of the treatment groups. **Figure 7A** shows that the body weights of the mice in the Epirubicin group and combination group were significantly lower (P=0.008; P=0.002) than those of the control. Apatinib did not significantly reduce body weights compared with the control group (P = 0.071). Peripheral blood samples were collected before sacrificing the mice and changes in biochemical indexes were evaluated. **Figure 7B** shows that compared with the control group, ALT (P = 0.011), UREA (P = 0.026), and LDH (P =0.021) increased in the Epirubicin group, UREA (P = 0.023) increased in the Apatinib group, and ALT (P = 0.011), AST (P = 0.029), and LDH (P = 0.008) increased in the combination group. H&E staining revealed no serious structural changes in the hearts, livers, or kidneys of the treated mice relative to the control group (**Figure S1**).

**Figure 8:**
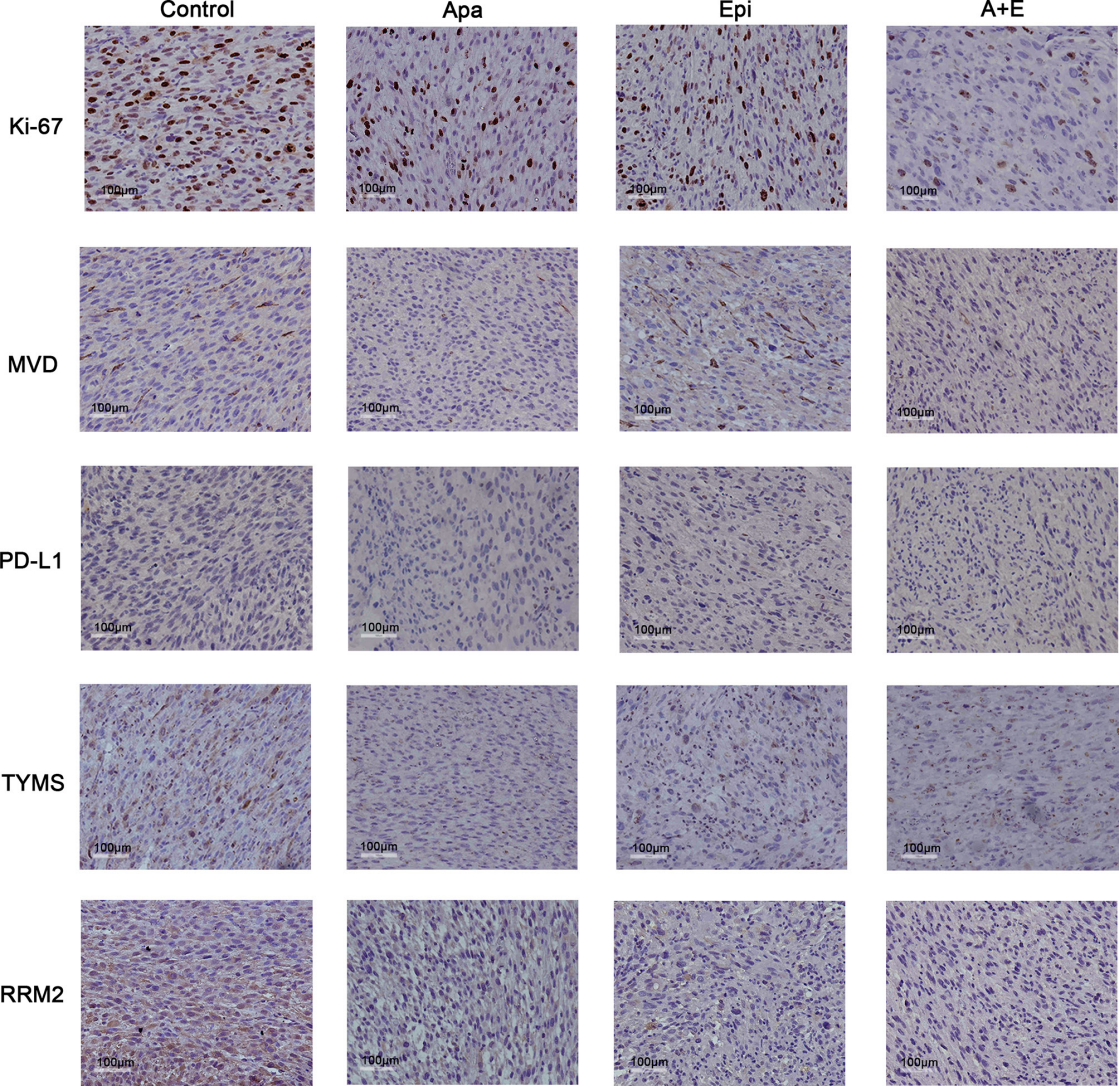
Apatinib decreased MVD and cell proliferation in liposarcoma PDX. IHC analysis of MVD, TYMS, MVD, RRM2, TYMS, Ki-67, and PD-L1 expression in tumor sections from each group. Compared with the control group, Apatinib could down-regulate MVD and the expression of TYMS and RRM2.The positive Ki-67 ratio decreased in all treatment groups. PD-L1 expression did not significantly differ among groups. ×200 magnification.

The IHC staining showed that MVD reduced in both Apatinib group and combination group. Hence, Apatinib might inhibit tumor growth *via* anti-angiogenic activity. The positive Ki-67 rates were lower in all treatment groups compared with the control group. RRM2 and TYMS were downregulated in the Apatinib group compared with the control group. PD-L1 expression did not significantly differ among groups (**Figure 8**).

## Discussion

Soft tissue sarcomas are heterogeneous solid tumors with unique biological features (19). The main treatment for these diseases is surgical resection. However, there is no efficacious therapy for postoperative recurrence of sarcoma or metastatic soft tissue sarcomas (STSs) (20). Hence, new therapeutic strategies for these tumors are urgently needed (4). Apatinib is a novel, small molecule anti-angiogenesis agent that inhibits VEGFR-2, c-kit, Src, and Ret. Phase II clinical trials have shown that Apatinib has manageable side effects and promising efficacy against various solid tumors (21–23). Two case studies suggested that Apatinib could benefit patients with liposarcoma (11, 12). Nevertheless, there is still relatively limited empirical or clinical evidence for the efficacy of Apatinib against liposarcoma. Thus, we conducted preclinical experiments on Apatinib treatment for liposarcoma.

Evaluation of MVD in tumor tissue is an important measure method of tumor angiogenesis. MVD is associated with TNM staging and prognosis in various tumors (24–26). Nevertheless, there are few reports on the relationship between MVD and clinicopathological features of STSs. The study of Virginia Baneth showed that MVD is higher in liposarcoma than the other four subtypes of sarcoma. Thus, liposarcoma might be relatively more sensitive to angiogenesis inhibitors (27). In the present study, the MVD of RLPS ranged between 12.5/mm^2^ to 225.0/mm^2^. For this reason, it may be important to screen patients who are likely to respond to anti-angiogenic drugs. A few studies showed that MVD is unsuitable as a prognosis predictor (28, 29). In our study, however, higher MVD was indicative of poorer OS in retroperitoneal liposarcoma patients (P = 0.024). Multivariate analysis also revealed that MVD is an independent risk factor for OS (HR: 1.974; 95%CI: 1.081–3.607; P = 0.001). These results suggested that MVD could be valuable in predicting the prognosis of retroperitoneal liposarcoma.

In subsequent cell experiments, Apatinib inhibited the proliferation, migration, and invasion of liposarcoma cells. Apatinib arrests the cell cycle at the G0/G1 phase. These were consistent with a prior study on the effect of Apatinib against osteosarcoma (9). Apatinib increased liposarcoma apoptosis in a concentration-dependent manner. The combination of Apatinib and Epirubicin more strongly inhibited SW872 and 93T449 cell proliferation than either individual drug. The results of animal experiments were similar to those for cell experiments. The combination of Apatinib and Epirubicin more strongly inhibited transplanted tumors in mice than either single drug alone. Our results suggested that the combination of these two drugs can synergically inhibit liposarcoma proliferation.

Our previous studies have indicated that TYMS and RRM2 are key genes in liposarcoma development and progression. Besides, our studies also found that TYMS and RRM2 mRNA expression was higher in liposarcoma than normal adipose tissues and that TYMS overexpression was associated with poor prognosis in liposarcoma patients. While the previous study revealed TYMS downregulation inhibited STAT3 phosphorylation in liposarcoma cell and RRM2 downregulation inhibited the AKT signaling pathway (30, 31). In the present study, RNA-seq and Western blot analysis showed that Apatinib might inhibit the RRM2/PI3K/AKT/mTOR signaling pathway and downregulate PD-L1 through the TYMS/STAT3 signaling pathway. Our results were consistent with those reported for thyroid cancer by Feng et al. and for non-small cell lung cancer by Liao (32, 33). Proliferation, invasion, and apoptosis are closely associated with AKT/mTOR signaling pathway (34). Therefore, we speculated that the antitumor function of Apatinib might be mediated by the RRM2/PI3K/AKT/mTOR signaling pathway.

Previous studies showed that STAT3 signaling pathway plays an important role in the inhibition of osteosarcoma proliferation by Apatinib. Moreover, inhibition of STAT3 signaling pathway can regulate PD-L1 expression (9, 35). Therefore, we assessed PD-L1 expression and changes in the STAT3 pathway in liposarcoma cell lines treated with Apatinib. Our results indicated that Apatinib did not upregulate PD-L1 as it did in the colorectal cancer (36); rather, it downregulated PD-L1 in liposarcoma. Hence, Apatinib might have different effects on tumor immunity in different tumor types. In lung cancer, low-dose Apatinib increases CD8+ T cell infiltration and has synergistic antitumor efficacy in combination with PD-1/PD-L1 immune checkpoint inhibitors (37). Yang et al. reported that PD-L1 promotes angiogenesis in ovarian cancer through the c-Jun/VEGFR-2 signaling axis. Furthermore, a combination of the PD-L1 inhibitor durvalumab plus Apatinib enhanced the anti-angiogenesis effect and inhibition of cell migration and invasion (38). These findings suggested that synergy between Apatinib and immunosuppressants does not merely involve a single pathway and that Apatinib may work synergistically with immunosuppressive agents in the treatment of retroperitoneal liposarcoma.

The thymidylate synthase TYMS plays an important role in DNA replication and repair. It also predicts patient sensitivity to 5-FU and pemetrexed (39, 40). However, RRM2 is closely associated with sensitivity to Adriamycin. Inhibition of pancreatic cancer cell lines by co-administration of Adriamycin and RRM2 siRNA was four times higher than that provided by Adriamycin administration alone (41).RRM2 overexpression upregulates VEGF in oral and pancreatic cancers and promotes tumor angiogenesis through the PI3K/AKT signaling pathway (42, 43). Certain TKIs may benefit patients with liposarcoma. However, prior reports mainly involved individual cases. A retrospective study on Anlotinib in West China Hospital revealed that sensitivity to this drug varied greatly among patients. Five patients had progression-free survival (PFS) < 10 wks, nine patients had PFS > 24 wks, and three patients had PFS between 10 wks and 24 wks (44). Therefore, biomarkers predicting patient sensitivity to TKI drugs are urgently needed. In our study, TYMS and RRM2 were inhibited after *in vivo* and *in vitro* Apatinib treatment. Hence, future research should endeavor to test TYMS and RRM2 as potential biomarkers for predicting Apatinib efficacy.

For liposarcoma, there currently lack effective treatment methods. Our results showed that Apatinib can inhibit the progression of tumors compared with the control group. In addition, in future studies, we will extend the administration time to observe further changes in tumors. We also observed that the tumors in the mice treated with Apatinib were pale in color and had some avascular necrosis. The current experiment is only a pre-clinical experiment. If it can be applied to the clinic in the future, we can apply Apatinib after the operation to delay the recurrence, and we can also use it in combination with immunotherapy to increase its therapeutic effect so that the tumor volume can be reduced.

To the best of our knowledge, the present study is the first to reveal the *in vitro* and *in vivo* antitumor effects of Apatinib against liposarcoma. As the incidence of retroperitoneal liposarcoma is relatively low, the number of patients enrolled in this study was still small. In future studies, more liposarcoma patients will be enrolled. The molecular mechanism underlying the synergistic effect between chemotherapeutic drugs and Apatinib is unclear. Future research will involve experiments in which liposarcoma cells and PDX models will be subjected to anlotinib, pazopanib, and other anti-angiogenic TKI drugs. In addition, we will attempt to elucidate the antitumor mechanisms of these drugs.

## Conclusions

Our research demonstrated that Apatinib alone and its combination with Epirubicin has a strong anti-tumor effect on liposarcoma *in vivo* and *in vitro*. Moreover, the combined use of Apatinib and epirubicin has a synergistic inhibitory effect. Apatinib may inhibit liposarcoma cell proliferation through RRM2/PI3K/AKT/mTOR signaling pathway, and down-regulate PD-L1 through TYMS/STAT3 signaling pathway.

## Data Availability Statement

The original contributions presented in the study are publicly available. This data can be found here: National Center for Biotechnology Information (NCBI) BioProject database under accession number GSE185783, https://www.ncbi.nlm.nih.gov/geo/query/acc.cgi?acc=GSE185783.

## Ethics Statement

The studies involving human participants were reviewed and approved by Institutional review board of Peking University Cancer Hospital (No. 2021KT43). The patients/participants provided their written informed consent to participate in this study. The animal study was reviewed and approved by Animal Ethics Committee of Peking University (No. EAEC2018-06).

## Author Contributions

Conception and design, CH, XT, and JW. Performing the experiments, LC, XT, JW, XG, LY, and DL. Evaluation of immunohistochemistry results, BD and MZ. Analysis and interpretation of data, XT, CH, AL, ZW, and FL. Writing of the manuscript, LC and XT. All authors contributed to the article and approved the submitted version.

## Funding

This study was supported by Capital Health Research and Development of Special Funds (approval No. 2020-1-1021), Beijing Municipal Natural Science Foundation (approval No.7153161 and Z190022), China Postdoctoral Science Foundation(approval No. 2020M680260), Beijing Municipal Administration of Hospital’s Ascent Plan (approval No. DFL20181104), Science Foundation of Peking University Cancer Hospital(approval No.2021-2, 2021-15, 2020-13 and 2020-14), Beijing Municipal Administration of Hospitals’ Youth Programme (approval No. QML20181104) and Beijing Excellent Talent Training Project (approval No.2018000021469G269).

## Conflict of Interest

The authors declare that the research was conducted in the absence of any commercial or financial relationships that could be construed as a potential conflict of interest.

## Publisher’s Note

All claims expressed in this article are solely those of the authors and do not necessarily represent those of their affiliated organizations, or those of the publisher, the editors and the reviewers. Any product that may be evaluated in this article, or claim that may be made by its manufacturer, is not guaranteed or endorsed by the publisher.
