# Supplementary material for: Anti-Tumor Effect of Apatinib and Relevant Mechanisms in Liposarcoma
Source: Front Oncol. 2021 Nov 18;11:739139. doi: 10.3389/fonc.2021.739139 (PMC8637299; doi:10.3389/fonc.2021.739139)
Supplement: Supplementary file 1 [file DataSheet_1.docx]

**Table S1 Top ten significantly up-regulated and down-regulated genes in SW872 cell line after apatinib application by RNA sequencing**

| Genes | log2 Fold change | P value | FDR |
| --- | --- | --- | --- |
| GPR1 | 4.124498 | 8.32E-07 | 4.55E-05 |
| FOSB | 3.826775 | 1.51E-16 | 1.38E-13 |
| FOS | 3.261243 | 3.16E-28 | 7.72E-25 |
| ASS1 | 3.021793 | 5.20E-17 | 5.08E-14 |
| PDGFB | 2.840538 | 2.03E-15 | 1.53E-12 |
| IL21R | 2.562573 | 1.75E-06 | 8.33E-05 |
| PTGDS | 2.509153 | 9.25E-07 | 4.93E-05 |
| SERPINE1 | 2.47176 | 6.15E-05 | 0.001308 |
| PCSK9 | 2.333967 | 3.52E-18 | 3.69E-15 |
| P2RX1 | 2.274769 | 0.000523 | 0.00663 |
| RN7SKP16 | -2.19425 | 7.65E-05 | 0.001528 |
| PI15 | -2.14063 | 1.71E-06 | 8.17E-05 |
| AC096921.2 | -2.1327 | 0.000134 | 0.002305 |
| FAM111B | -2.05332 | 0.001048 | 0.011215 |
| AC025176.1 | -1.91686 | 0.001422 | 0.01377 |
| DOK3 | -1.8653 | 2.04E-08 | 2.22E-06 |
| AC114956.2 | -1.8646 | 0.00029 | 0.004158 |
| RASSF6 | -1.82572 | 0.001164 | 0.011979 |
| CHRNA10 | -1.77616 | 0.004684 | 0.032303 |
| CCNE2 | -1.72284 | 1.83E-08 | 2.09E-06 |

FDR: False discovery rate

Figure S1


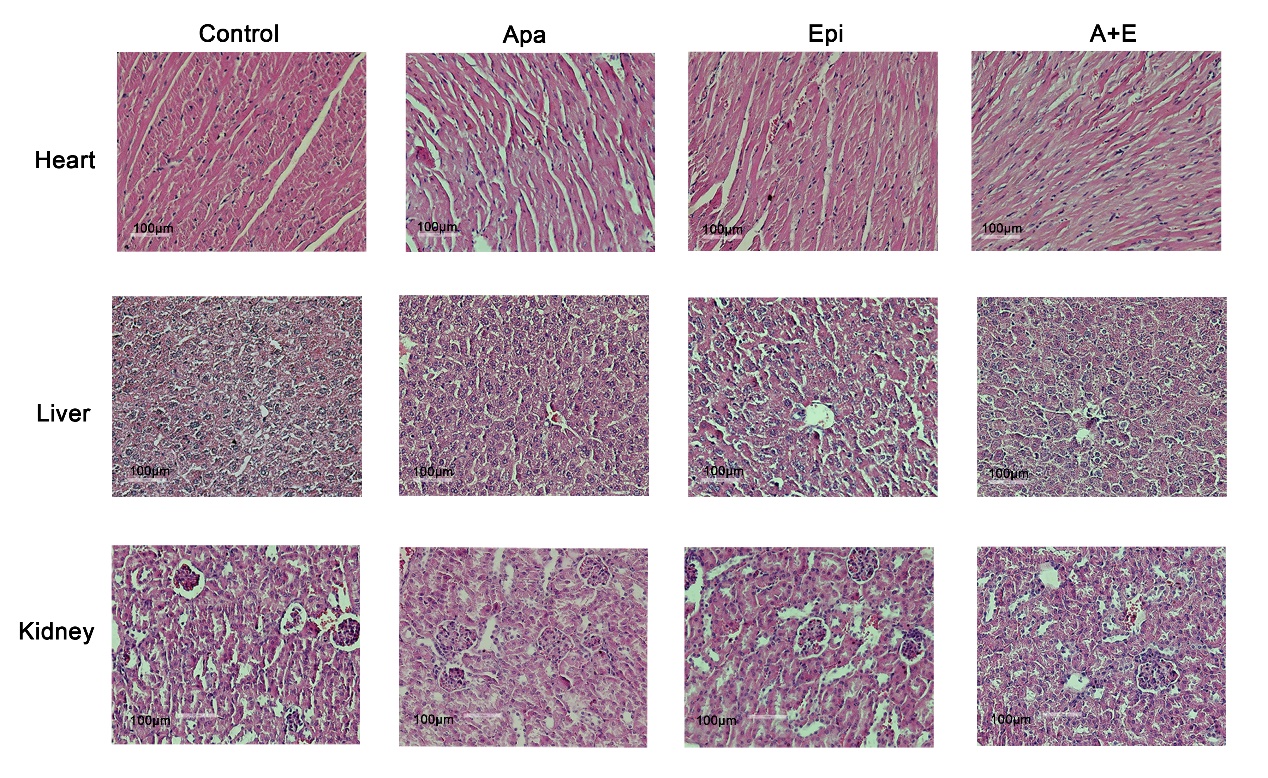


H&E staining of liver, heart, and kidney sections in each mice group. No significant changes in organ structure were observed in each group.
